# Exploring the molecular mechanism of Si-Miao-Yong-An Decoction in treating diabetic foot using network pharmacological analysis and molecular docking technique

**DOI:** 10.1097/MD.0000000000042629

**Published:** 2025-05-30

**Authors:** Qiang Zhang, Jing Li, Lin Wang, Qiu-ying Zhang, Ji Guan

**Affiliations:** a Department of Chinese Medicine, The Fifth People’s Hospital of Ningxia Hui Autonomous Region, Shizuishan, Ningxia, China; b Surgical oncology, The Fifth People’s Hospital of Ningxia Hui Autonomous Region, Shizuishan, Ningxia, China.

**Keywords:** diabetic foot, diabetic foot ulcer, molecular docking, network pharmacological analysis, Si-Miao-Yong-An Decoction

## Abstract

This study explores the potential mechanism of Si-Miao-Yong-An Decoction (SMYAD) in the treatment of diabetic foot using network pharmacological and molecular docking. The SMYAD- and diabetic foot ulcer (DFU)-related targets were obtained from traditional Chinese medicine systems pharmacology database, GeneCards and DisGeNET. The common targets and compounds were determined by Venn diagram. Then, protein–protein interaction network was constructed in STRING and Cytoscape, and MCODE plugins was used to identify the cluster and hub genes. The differentially expressed hub genes were determined using limma R package. In addition, the gene ontology and Kyoto Encyclopedia of Genes and Genomes enrichment was performed in R software. Autodock Vina and PyMOL were applied for molecular docking. In total, 401 common targets of SMYAD-related targets and DFU-related targets and 104 active components related to common targets were identified. Besides, 10 hub genes, including cyclin dependent kinase 4, ataxia telangiectasia mutated, mitogen-activated protein kinase 3, janus kinase 1, histone deacetylase 1, fibroblast growth factor 2, matrix metallopeptidase 9, platelet derived growth factor receptor beta, cyclin D1, and peroxisome proliferator activated receptor gamma, were obtained through differentially expressed gene analysis. The key active components of SMYAD in the treatment of DFU were 7,2′,4′-trihydroxy-5-methoxy-3-arylcoumarin, licochalcone B, glyasperin F, vestitol, glyasperins M and (2R)-7-hydroxy-2-(4-hydroxyphenyl)chroman-4-one. PI3K-Akt signaling pathway and advanced glycation end product-receptor of advanced glycation end product signaling pathway in diabetic complications were the key signal pathways for SMYAD in the treatment of DFU. The results of molecular docking showed that some key active components were well connected with hub gene. This study reveals the potential mechanism of SMYAD in the treatment of DFU through multi-components, multi-targets and multi-channels, which provides more theoretical basis for the study of SMYAD’s mechanism.

## 1. Introduction

Diabetes has become a global chronic metabolic disease, with an increasing incidence among adults.^[1]^ Diabetic foot ulcer (DFU), which is mainly caused by pathological damage of blood vessels and nerves, is one of the main chronic complications of diabetes, and it leads to the 14% to 24% amputation rate of lower limbs of patients.^[2–4]^ Currently, the main treatments of DFU in western medicine include lowering blood sugar, improving blood vessels, anti-infection, cleaning wounds and surgical treatment.^[5]^ Despite the fact that these treatment approaches can effectively manage infection, restore the blood flow function of blood vessels and accelerate wound healing, their effectiveness varies greatly, leading to persistently high rates of amputation and mortality.^[6]^ In recent years, people have realized that traditional Chinese medicine (TCM) has the advantages of minor side effects and remarkable curative effects, which leads to its wide application in enhancing human immunity, preventing and treating infectious and chronic diseases.^[7]^ In the theoretical system of TCM, DFU belongs to the category of “gangrene.” It is reported that TCM can improve DFU through maintaining inflammatory balance, inhibiting oxidative stress and angiogenesis.^[8]^

Si-Miao-Yong-An Decoction (SMYAD) was first recorded in “YanFangXinBian,” in which herbs include Xuan-Shen (Figwort Root), Jin-Yin-Hua (Lonicerae Japonicae Flos), Dang-Gui (Angelicae Sinensis Radix), and Gan-Cao (licorice).^[9]^ Jin-Yin-Hua is useful for the heat clearing and detoxifying, and Xuan-Shen has the effect of nourishing yin and reducing fire, and Dang-Gui commonly used in activating blood circulation and dissipating blood stasis, and Gan-Cao has the functions of promoting dendritic cell maturation, regulating the secretion of inflammatory cytokines, exerting immune regulation and anti-inflammation.^[9,10]^ It has been reported that SMYAD has a good effect in treating diabetic retinopathy and improving the cardiac function of diabetic mice.^[11]^ However, the mechanism of SMYAD in treating DFU remains unclear. Therefore, exploring the mechanism of SMYAD in the treatment of DFU could provide new options for the treatment of DFU.

In this study, the network pharmacology method and molecular docking technology were used to identify the hub gene mainly involved in the treatment of DFU by SMYAD and explore the potential mechanism. Firstly, we screened out the effective active ingredients, common targets, hub gene, and key active ingredients that may be involved in the treatment of DFU by SMYAD. Secondly, key signal pathways were screen out using enrichment analysis. Finally, the interaction between key active components and hub gene was verified by molecular docking.

## 2. Materials and methods

### 2.1. Screening of compound components and targets in SMYAD

At first, we obtained the active ingredients related to Xuan-Shen, Jin-Yin-Hua, Gan-Cao, and Dang-Gui from the traditional Chinese medicine systems pharmacology database (TCMSP, https://old.tcmsp-e.com/tcmsp.php). Then, the active ingredients were determined based on oral bioavailability (OB)≥ 30% and drug-likeness≥ 0.18 conditions.^[12]^ OB≥ 30% indicates that the compound has high oral availability and slow metabolism, while drug-likeness≥ 0.18 suggests that the compound has good efficacy in drug development.^[13,14]^ Subsequently, the Molecule IDs of the active ingredients were used to determine their Canonical SMILES in the PubChem database. Finally, we predicted the drug targets of the active ingredients using Canonical SMILES in the Swiss Target Prediction database.

### 2.2. Acquisition of DFU-related targets

The DFU-related target genes were obtained through searching for “DFU” in GeneCards (https://www.genecards.org) and DisGeNET (https://www.disgenet.org/home/) databases. Then, the 2 datasets of DFU-related genes were combined to form a set of DFU-related target genes.

### 2.3. Screening of hub genes

To begin with, the common targets were imported into the STRING (https://cn.string-db.org/) to obtain protein-protein interaction (PPI) information. Then, the isolated proteins were removed and the search criteria was set as “Homo sapiens” with a confidence score≥ 0.4. Next, we visualized the PPI information using Cytoscape software. Afterwards, the cytoHubba plugin was used to calculate the topological features of the PPI network and adjust the size and color of nodes based on the degree in Cytoscape. Finally, we used the MCODE plugin in Cytoscape to cluster the important modules in the PPI network and selected the most significant module for further analysis. The advanced options were set as Degree cutoff= 2, Node Score Cutoff= 0.2, and K-Core= 2.

Furthermore, we identified the differentially expressed genes in the most significant module using the GSE29221 dataset. The inclusion criteria for the dataset were a sample size of no <20 cases, and the samples should include both patients with DFU and those without DFU.

### 2.4. Screening of key active ingredients

The top 10 effective active ingredients with OB≥ 30% were selected as important active ingredients. In addition, the intersection of important active ingredients and hub gene-corresponding active ingredients were screened out as key active ingredients using the Venn diagram.

### 2.5. Screening of key signal pathways

The gene ontology (GO) and Kyoto Encyclopedia of Genes and Genomes (KEGG) were applied for enrichment analysis using the clusterProfile package in R software based on DFU-related targets and common targets. The results of GO analysis describe the functions of gene products from 3 aspects: biological process, cellular component, and molecular function. KEGG is a database that integrates genomic, chemical, and systemic functional information. Then, the top 10 significant signal pathways in the KEGG enrichment were determined among which associated with DFU were considered as key signal pathways.

### 2.6. The construction of interaction network

After the interactions between the herbs, effective active ingredients, common targets, signal pathways and DFU were sorted and imported into Cytoscape, the relationship network was visualized. In this network, nodes represent herbs, active ingredients and common targets, and edges represent their relationships.

### 2.7. Molecular docking

The key active ingredients and hub genes in SMYAD were selected for the molecular docking. The 3D structure files of key active ingredients were downloaded in mol2 format from the TCMSP database based on their Molecule IDs. Then, the 3D structure files of hub genes in PDB format with higher resolution and fewer ligands were obtained from the RCSB PDB database (https://www.rcsb.org/). Furthermore, the crystal structure of the hub gene was processed through removing water molecules, hydrogenation and excess protein chains in AutoTools software, and it was set as the receptor. In addition, the key active ingredients were hydrogenated and checked for torsional bonds and set as ligands. Besides, AutoDock (Version 1.5.6) was used to simulate the docking state between proteins and small molecules. A binding energy less than −5.0 kcal/mol was considered to indicate a good binding between ligands and receptors.^[15]^ Finally, the docking results were visualized using PyMOL software.

## 3. Results

### 3.1. The effective components and target of SMYAD

In order to obtain the active ingredients and target genes of SMYAD, we screened the active ingredients of SMYAD using the TCMSP database and predicted the target genes related to the active ingredients using the Swiss Target Prediction database. The results showed that there were 2 active ingredients and 44 target genes in Dang-Gui, 92 active ingredients and 784 target genes in Gan-Cao, 23 active ingredients and 419 target genes in Jin-Yin-Hua, and 9 active ingredients and 172 target genes in Xuan-Shen. Ultimately a total of 120 active ingredients and 879 drug targets were obtained (Table 1).

**Table 1:** The effective components and target of SMYAD.

SMYAD	Ingredients	Targets
Dang-Gui	2	44
Gan-Cao	92	784
Jin-Yin-Hua	23	419
Xuan-Shen	9	172

SMYAD = Si-Miao-Yong-An Decoction.

### 3.2. The selection of DFU-related target genes

We obtained 3001 and 146 DFU-related targets using the CeneCards and DisGeNET databases, respectively. After merging the DFU-related targets and removing duplicate targets, 3041 DFU-related targets were finally obtained.

### 3.3. The construction of TCM-active ingredients-common targets interaction network

**Figure 1.**
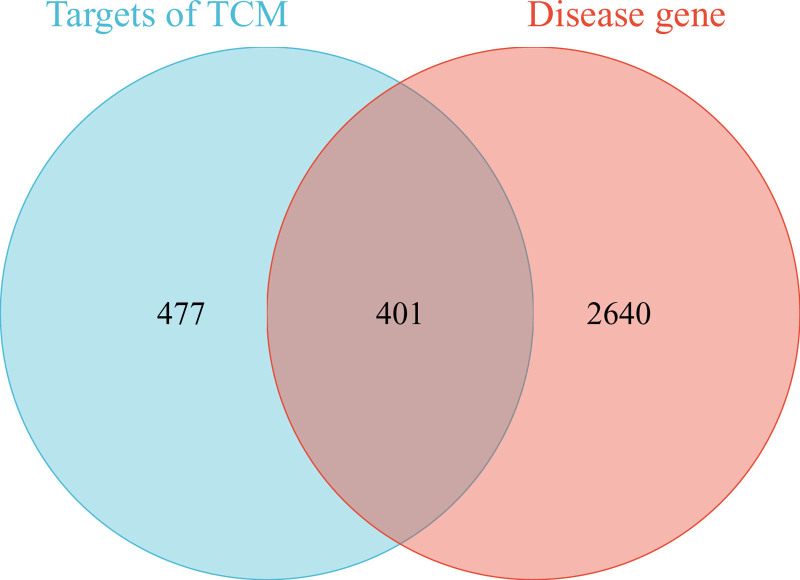
The Venn diagram of the SMYAD and DFU related target genes. DFU = diabetic foot ulcer, SMYAD = Si-Miao-Yong-An Decoction.

**Figure 2.**
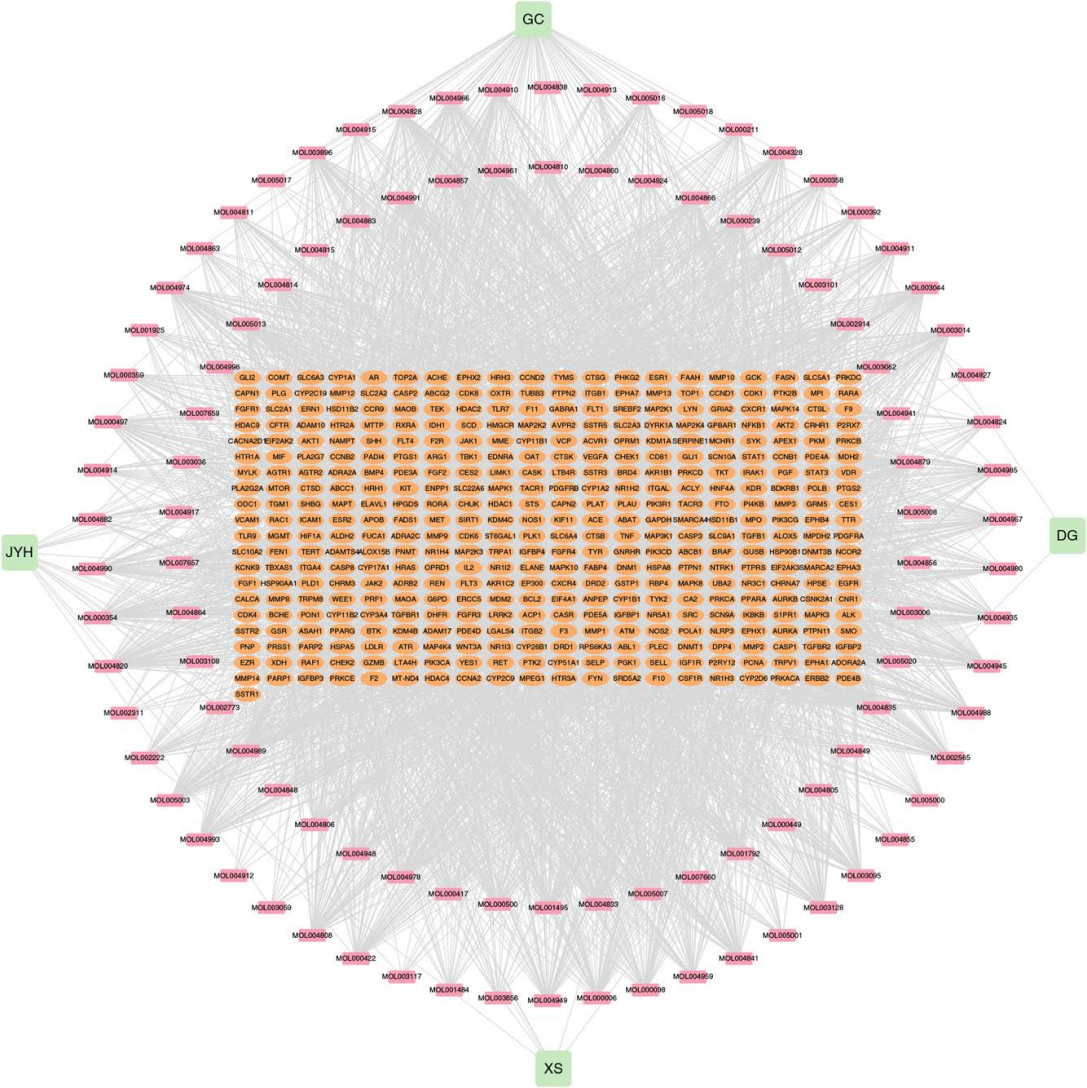
The TCM-active ingredients-common targets interaction network. TCM = traditional Chinese medicine.

Firstly, 401 common targets between SMYAD and DFU were obtained through Venn diagram (Fig. 1). Secondly, we constructed an interaction network of TCM-active ingredients-common targets. The network exhibited that SMYAD was mainly involved in treating DFU through 104 effective active ingredients and 401 common targets (Fig. 2).

### 3.4. The identification of hub genes

**Figure 3.**
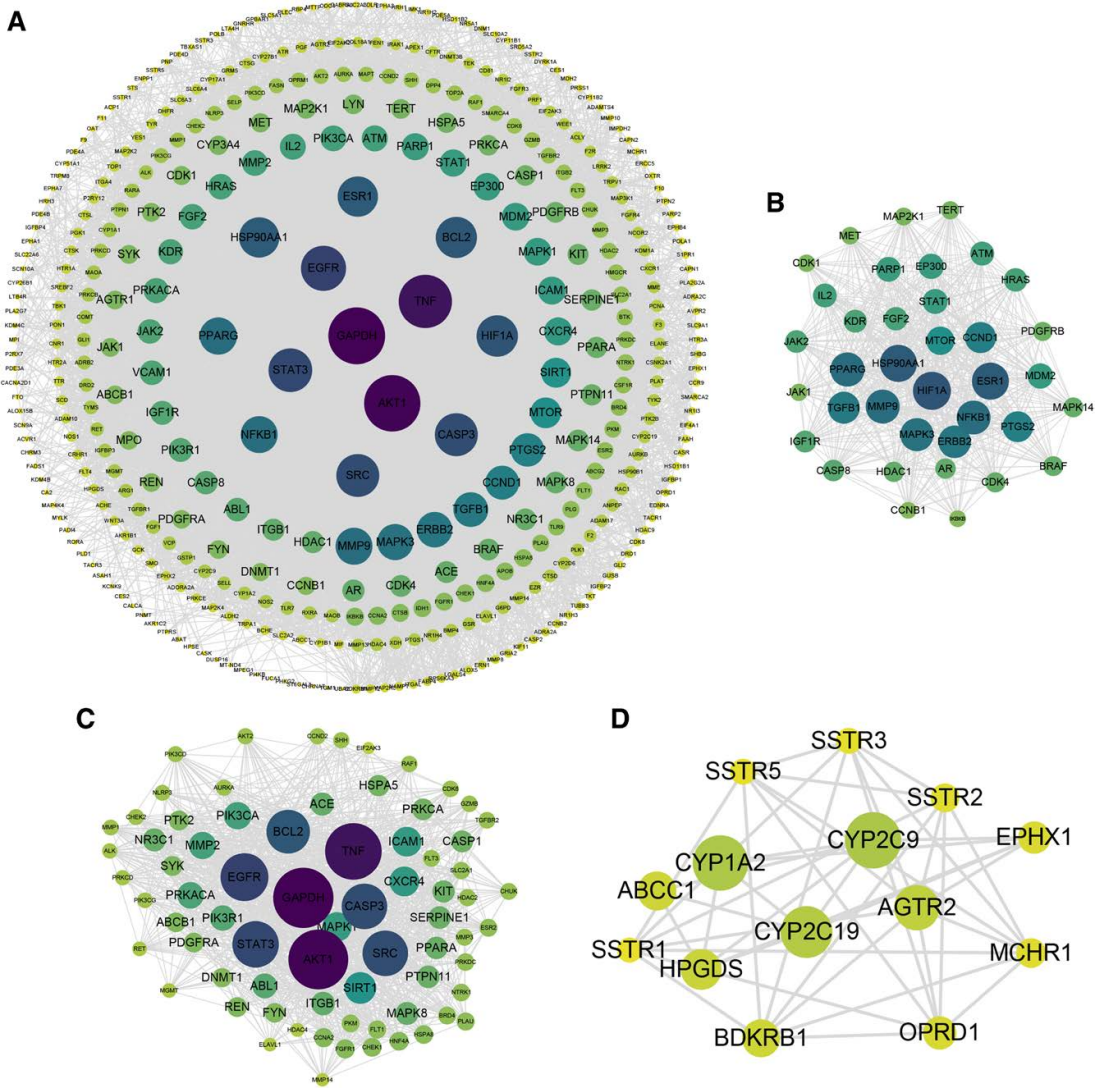
The clustering analysis of common targets. (A) PPI network of common targets. The size and color depth of nodes represented degree. (B–D) The top 3 modules with the highest scores in the clustering analysis results of common targets. PPI = protein–protein interaction.

To obtain the hub genes in SMYAD for DFU treatment, after 401 common targets were first imported into the STRING database for PPI analysis and the free nodes were removed, the results were visualized using Cytoscape software. As shown in Figure 3A, there were a total of 386 nodes and 8817 edges in the PPI network. In addition, we used the MCODE plugin in Cytoscape to cluster the targets and obtained 12 modules, and the top 3 modules with the highest module scores were presented in Figure 3B–D. Each module score represented the core density of nodes and adjacent nodes in the topology, and the higher module score represented the more concentrated the clustering.^[16]^ Thus, we further validated the differential expression of the 37 genes from the module with the highest scores using the GSE29221 dataset, and obtained 10 hub genes, including cyclin dependent kinase 4 (CDK4), threonine kinase (ataxia telangiectasia mutated [ATM]), mitogen-activated protein kinase 3 (MAPK3), janus kinase 1(JAK1), histone deacetylase 1 (HDAC1), fibroblast growth factor 2 (FGF2), matrix metallopeptidase 9 (MMP9), platelet derived growth factor receptor beta (PDGFRB), cyclin D1 (CCND1) and peroxisome proliferator activated receptor gamma (PPARG).

### 3.5. The determination of key active ingredients

Initially, the top 10 effective active ingredients with the highest OB from the 104 effective active ingredients were selected (Table 2). Then, the intersection of 10 important active ingredients and 71 active ingredients related to hub genes were screened out as key active ingredients using the Venn diagram. The key active ingredients include MOL004990, MOL004841, MOL004810, MOL000500, MOL005007, and MOL004941 (Table 3).

**Table 2:** The top 10 active components of OB among the active components related to common targets.

Mol ID	Molecule name	OB	DL
MOL002311	Glycyrol	90.78	0.67
MOL003006	(−)-(3*R*,8*S*,9*R*,9a*S*,10a*S*)-9-ethenyl-8-(beta-d-glucopyranosyloxy)-2,3,9,9a,10,10a-hexahydro-5-oxo-5H,8H-pyrano[4,3-d]oxazolo[3,2-a]pyridine-3-carboxylic acid_qt	87.47	0.23
MOL004990	7,2′,4′-trihydroxy-5-methoxy-3-arylcoumarin	83.71	0.27
MOL005017	Phaseol	78.77	0.58
MOL004841	Licochalcone B	76.76	0.19
MOL004810	glyasperin F	75.84	0.54
MOL001484	Inermine	75.18	0.54
MOL000500	Vestitol	74.66	0.86
MOL005007	Glyasperins M	72.67	0.59
MOL004941	(2*R*)-7-hydroxy-2-(4-hydroxyphenyl)chroman-4-one	71.12	0.18

DF = drug-likeness, OB = oral bioavailability.

**Table 3:** The active components related to the hub gene.

CDK4		MMP9		HDAC1	PPARG		PDGFRB	CCND1	MAPK3	JAK1	ATM	FGF2
MOL004991	MOL004835	MOL005012	MOL003095	MOL005020	MOL004820	MOL004989	MOL005012	MOL004835	MOL003128	MOL004833	MOL004974	MOL001925
MOL004848	MOL004841	MOL004328	MOL004935	MOL005003	MOL001792	MOL004985	MOL001495	MOL002565	MOL005013	MOL003014		
MOL004993	MOL004828	MOL004993	MOL000006	MOL004988	MOL000359	MOL003128	MOL005007	MOL004914	MOL000211	MOL004824		
MOL004978	MOL005003	MOL004835	MOL004990	MOL004808	MOL002914	MOL004808	MOL004811	MOL005007	MOL004996			
MOL005007	MOL003014	MOL000497	MOL004948	MOL000497	MOL004993	MOL000358	MOL004924	MOL004864	MOL004985			
MOL004989	MOL004957	MOL004961	MOL003044	MOL004991	MOL004935	MOL005000	MOL004991	MOL004974				
MOL000500	MOL004856	MOL004824	MOL004989	MOL004966	MOL004948	MOL004941	MOL004914	MOL004848				
MOL004911	MOL004913	MOL003014	MOL000239	MOL004820	MOL000211	MOL007657	MOL004841	MOL004806				
MOL004980	MOL005012	MOL004879	MOL004814	MOL004810	MOL003896	MOL004806	MOL004913	MOL004978				
MOL004864	MOL002565	MOL000422	MOL004866	MOL004841	MOL000449	MOL004328	MOL004808	MOL004828				
MOL004849	MOL004806	MOL000354	MOL000098	MOL003117	MOL004866	MOL001495	MOL003896	MOL004911				
MOL002222	MOL004941	MOL001792		MOL004974	MOL004945		MOL000497	MOL004957				
MOL004328		MOL004820		MOL004835	MOL005013		MOL004988	MOL003014				
MOL004974		MOL002914		MOL004879	MOL004882		MOL003128	MOL004849				
MOL004914		MOL004828		MOL004957	MOL004910		MOL007657					
MOL004966		MOL004911		MOL005013	MOL004996		MOL004833					

ATM = ataxia telangiectasia mutated, CCND1 = cyclin D1, CDK4 = cyclin dependent kinase 4, FGF2 = fibroblast growth factor 2, HDAC1 = histone deacetylase 1, JAK1 = janus kinase 1, MAPK3 = mitogen-activated protein kinase 3, MMP9 = matrix metallopeptidase 9, PDGFRB = platelet derived growth factor receptor beta, PPARG = peroxisome proliferator activated receptor gamma.

### 3.6. The GO and KEGG enrichment analysis

**Figure 4.**
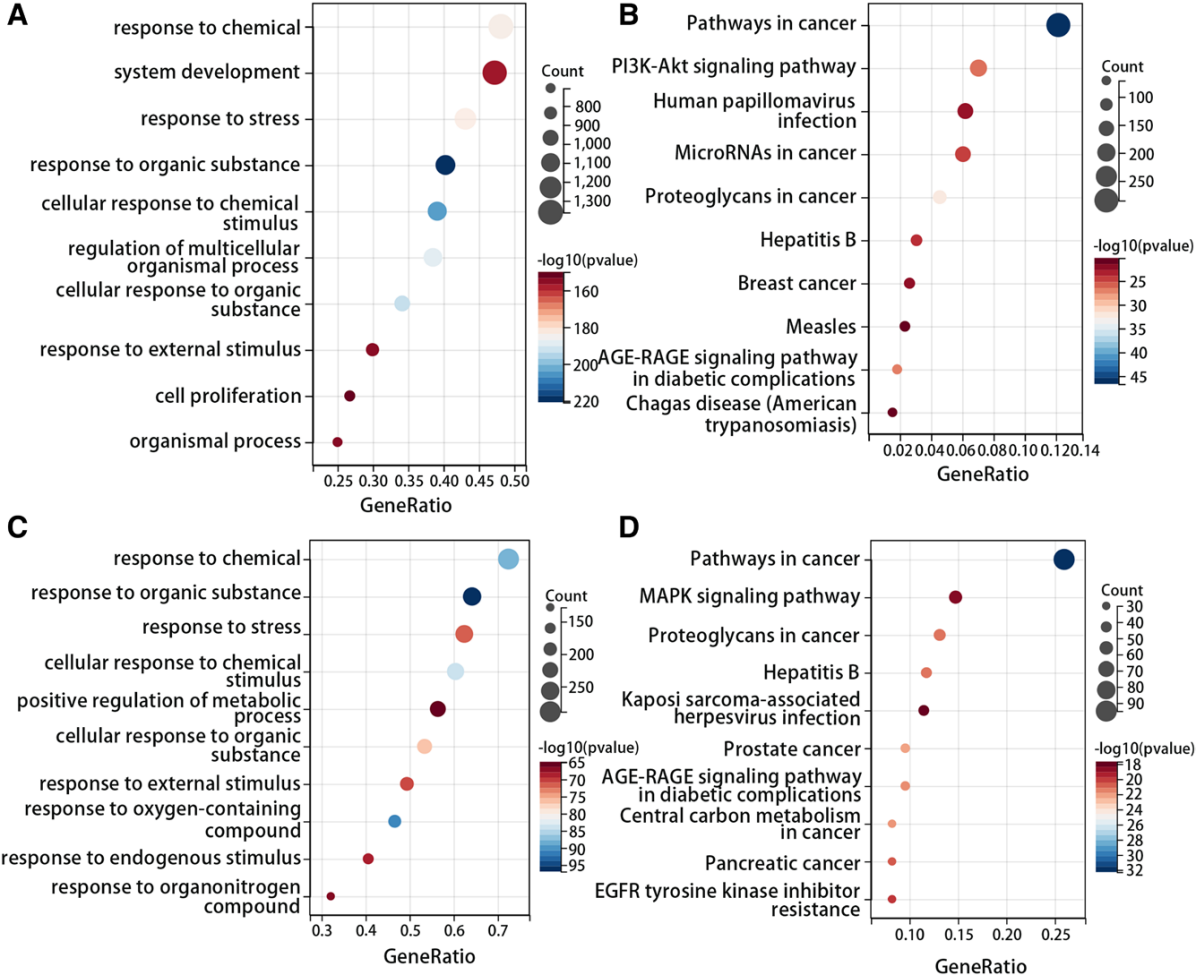
Gene Ontology (GO) and Kyoto Encyclopedia of Genes and Genomes (KEGG) analysis of DFU-related targets and common targets. (A) Top 10 GO terms of DFU-related targets. (B) Top 10 signal pathways in KEGG pathway enrichment of DFU-related targets. (C) Top 10 GO terms of common targets. (D) Top 10 signal pathways in KEGG pathway enrichment of common targets. DFU = diabetic foot ulcer , GO = gene ontology, KEGG = Kyoto Encyclopedia of Genes and Genomes.

We respectively performed enrichment analysis on DFU-related targets and common targets to screen the key signal pathways in SMYAD for DFU treatment. The GO analysis showed that DFU-related targets were mainly enriched in response to chemical, system development, response to stress, response to organic substance, and cellular response to chemical stimulus (Fig. 4A). The KEGG pathway analysis revealed that DFU-related targets mainly participated in pathways in cancer, phosphatidylinositide 3-kinases (PI3K)-protein kinase B (Akt) signaling pathway, human papillomavirus infection, and advanced glycation end product (AGE)-receptor of AGE (RAGE) signaling pathway in diabetic complications (Fig. 4B). Besides, the GO analysis of common targets showed that they were mainly enriched in response to chemical, response to organic substance, response to stress, and cellular response to chemical stimulus (Fig. 4C). The KEGG pathway enrichment of common targets indicated that they were mainly involved in pathways in cancer, MAPK signal pathway, proteoglycans in cancer, and AGE-RAGE signal pathway in diabetic complications (Fig. 4D).

According to the results of KEGG enrichment based on DFU-related targets and common targets, PI3K-Akt signaling pathway and AGE-RAGE signaling pathway in diabetic complications were considered as the key signal pathways.

### 3.7. The construction of SMYAD-herbs-active ingredients-hub gene-signal pathway-DFU signal pathway interaction network

**Figure 5.**
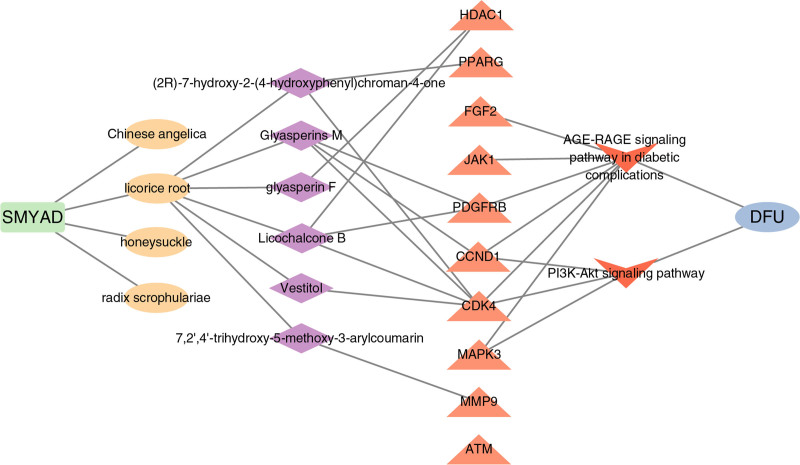
SMYAD-herbs-active ingredients-hub gene-signal pathway-DFU signal pathway interaction network. Light green square nodes represent SMYAD, light yellow oval nodes represent traditional Chinese medicine, purple diamond nodes represent active ingredients, red triangle nodes represent hub gene, dark red arrow nodes represent signal pathways and light blue oval nodes represent DFU. DFU = diabetic foot ulcer, SMYAD = Si-Miao-Yong-An Decoction.

To explore the potential mechanism of SMYAD in the treatment of DFU, a SMYAD-herb-active ingredient-hub gene-signaling pathway-DFU interaction network were constructed. As shown in Figure 5, this network included 24 nodes and 32 edges.

### 3.8. The verification of results by molecular docking

The molecular docking simulations was used to explore the interaction between key active ingredients and hub genes in the SMYAD-herb-active ingredient-hub gene-signaling pathway-DFU network. After the 3D structures of CCND1, CDK4, HDAC1, MMP9, PDGFRB, and PPARG were obtained in the PDB database, AutoDock was applied for molecular docking.

**Figure 6.**
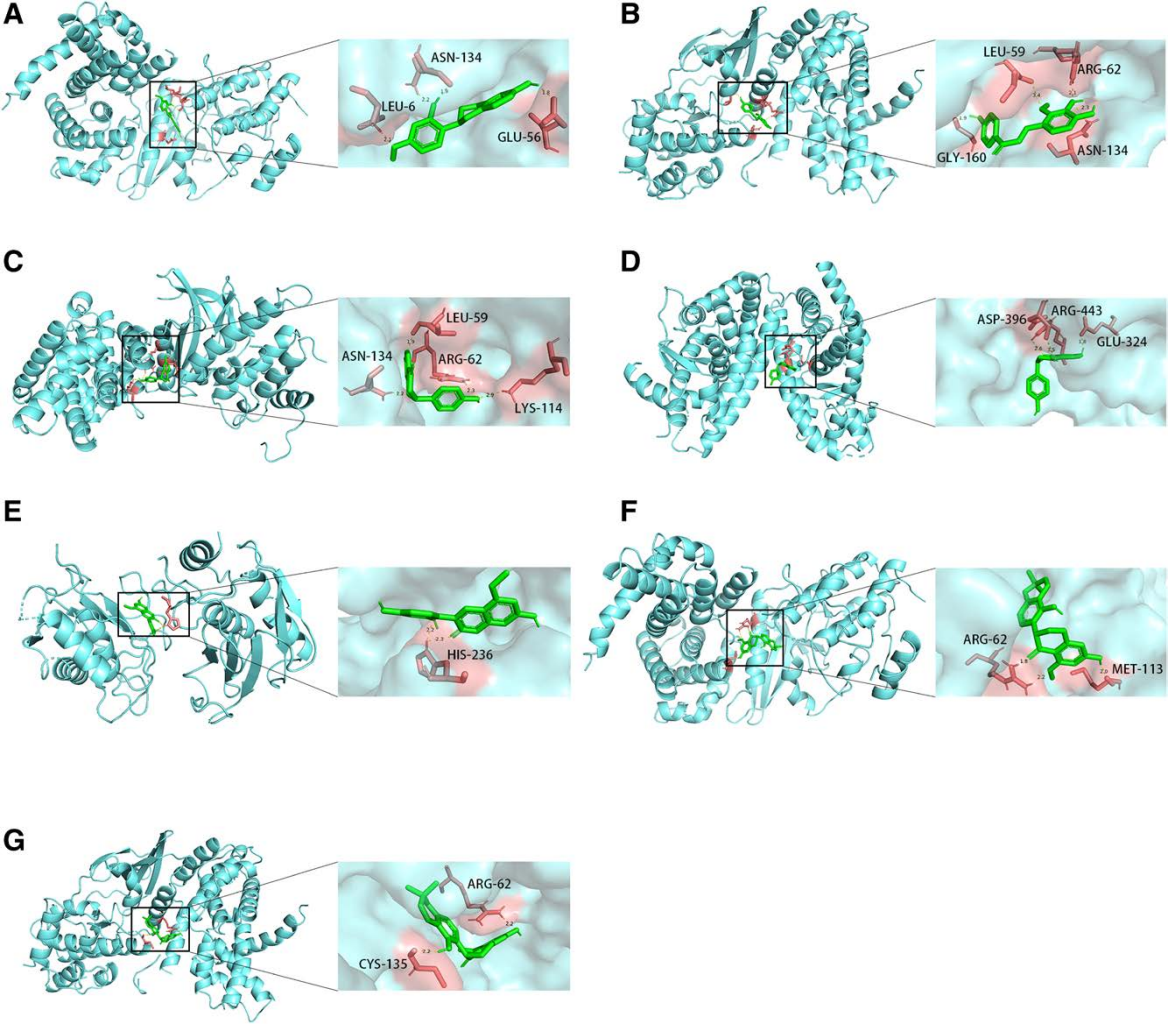
The molecular docking of key active ingredients and hub genes. (A) The molecular docking of MOL000500 and CDK4. (B) The molecular docking of MOL004841 and CDK4. (C) The molecular docking of MOL004941 and CDK4. (D) The molecular docking of MOL004941 and PPARG. (E) The molecular docking of MOL004990 and MMP9. (F) The molecular docking of MOL005007 and CCND1. (G) The molecular docking of MOL005007 and CDK4. CCND1 = cyclin D1, CDK4 = cyclin dependent kinase 4, MMP9 = matrix metallopeptidase 9, PPARG = peroxisome proliferator activated receptor gamma.

The docking results showed that some results were excellent (Table 4). Figure 6 revealed that the best docking results between key active ingredients and corresponding proteins of hub genes. Each small molecule was able to enter the active pocket of the protein, indicating a good matching characteristic.^[16]^

**Table 4:** Molecular docking binding energy of key active components in SMYAD with hub gene.

Active ingredients	Molecule name	Hub targets (PDB ID)	Binding energy (kcal/mol)
MOL000500	Vestitol	CDK4(2w96)	−6.6
MOL004841	Licochalcone B	CDK4(2w96)	−7.11
MOL004941	(2*R*)-7-hydroxy-2-(4-hydroxyphenyl)chroman-4-one	CDK4(2w96)	−5.78
MOL004941	(2*R*)-7-hydroxy-2-(4-hydroxyphenyl)chroman-4-one	PPARG(4ci5)	−4.88
MOL004990	7,2′,4′-trihydroxy-5-methoxy-3-arylcoumarin	MMP9(4h1q)	−5.53
MOL005007	Glyasperins M	CCND1(2w96)	−6.16
MOL005007	Glyasperins M	CDK4(2w96)	−6.29

SMYAD = Si-Miao-Yong-An Decoction.

## 4. Discussion

Diabetes foot (DF) is a peripheral vascular neuropathy caused by microcirculatory disorders and pathological changes in small blood vessels of the lower extremities in diabetic patients, which presents with bilateral foot pain, coldness, and numbness.^[17]^ Studies have shown that DFU is a disease with high incidence and recurrence rates, with a lifetime incidence of 19% to 34% and a recurrence rate within 1 year after treatment of 40%, and a recurrence rate within 3 years of 65%.^[18]^ Currently, the basic treatment for DFU patients in clinical practice mainly includes comprehensive treatments such as controlling blood glucose, local debridement and changing dressings, and anti-infection, but its efficacy is limited and the mechanism is unclear.^[19]^ Previous studies have shown that SMYAD, a classic Chinese herbal formula, had satisfactory effects on anti-inflammation, anticoagulation, lipid reduction and vessel protection, among which the anti-inflammatory effect is particularly prominent.^[20]^ In order to explore the potential mechanism of SMYAD in the treatment of DFU, the key effective active ingredients, hub genes, and key signaling pathways in SMYAD were screened out based on network pharmacology analysis and molecular docking technology.

DF is a complex pathological process closely related to neurological and microvascular damage caused by oxidative stress reactions and inflammatory responses.^[21]^ Our results revealed that the hub genes of SMYAD in the treatment of diabetic foot included CDK4, ATM, MAPK3, JAK1, HDAC1, FGF2, MMP9, PDGFRB, CCND1, and PPARG. CDK4 can control the main stages of the cell cycle from growth (G1) to DNA replication (S) phase.^[22]^ Gene knockout experiments identified that mice with knockout alleles of CDK4 develop diabetic phenotypes, including a 90% reduction in blood glucose levels, polyuria, polydipsia, rapid volume loss and a sharp decrease in pancreatic *β*-cells.^[23]^ MAPK3 is not only closely related to diabetic heart failure but may also be an important gene causing vascular damage in type 2 diabetes.^[24,25]^ Some studies reported that JAK1, one of the main members of the JAK family, was closely related to inflammatory pathways. Blocking the inflammatory pathway mediated by JAK1 can change the innate and adaptive immune responses involved in inflammatory bowel disease, thereby reducing chronic gastrointestinal inflammation.^[26]^ HDAC1 could regulate endothelial function, mainly involving angiogenesis, inflammatory signal transduction, redox homeostasis, and NO production.^[27]^ FGF2 is a member of the FGF family and can promote the development of ovarian through mediating cell proliferation, differentiation, and apoptosis.^[28]^ In diabetic patients, persistently high blood glucose levels could cause oxidative stress and induce the synthesis of MMP9 which was involved in various cellular processes, including angiogenesis and neurogenesis.^[29]^ PDGFRB could encode the receptor for platelet derived growth factor beta, and it was expressed in neurons, smooth muscle cells, and pericytes.^[30]^ MicroRNA-532-5p could regulate the oxidative stress and insulin secretion damage induced by high glucose in pancreatic β cells by downregulated expression of CCND1.^[31]^ The PPARG gene encoded PPAR-γ which was necessary for maintaining lipid and glucose homeostasis.^[32]^ PPAR-γ in adipocytes could maintain the secretion of adipocytokines, which helped to mediate the function of insulin in peripheral tissues and maintained insulin sensitivity.^[33]^ Therefore, we inferred that SMYAD may treat DFU by targeting CDK4, ATM, MAPK3, JAK1, HDAC1, FGF2, MMP9, PDGFRB, CCND1, and PPARG.

Network pharmacology analysis showed that the key active ingredients of SMYAD in the treatment of DFU included 7,2′,4′-trihydroxy-5-methoxy-3-arylcoumarin, licochalcone B, glyasperin F, vestitol, glyasperins M and (2R)-7-hydroxy-2-(4-hydroxyphenyl) chroman-4-one. Previous studies have shown that arylcoumarins have various activities, including anticancer, antileukemia, anticoagulation, anti-inflammatory, and antioxidant effects.^[34]^ In genetic diabetic mice, 3-arylcoumarin can significantly reduce blood glucose levels.^[35]^ Licochalcone B has antidiabetic effects in Uralobacterium sp. and could be used as a functional food ingredient for controlling type 2 diabetes.^[36]^ A study has shown that glyasperin F could regulate cell growth by inhibiting the activation of MMP1, thereby reducing tissue damage and cell death caused by excessive inflammatory responses.^[37]^ Vestitol is a flavonoid compound and it has been proven to have significant anti-inflammatory effects.^[38]^ Thus, SMYAD might exert pharmacological effects on DFU through the above active ingredients.

Our results demonstrated that the PI3K-Akt signaling pathway and AGE-RAGE signaling pathway in diabetic complications may be the key signal pathways for SMYAD to treat DFU. The PI3K/Akt signal pathway is an important node in insulin signaling, which can regulate glucose uptake, cellular metabolism, cell survival, proliferation, migration, and glycogen synthesis.^[39]^ Insulin could regulate beta cell function and insulin secretion through the PI3K/Akt pathway, and pancreatic beta cells can produce and secrete insulin in response to blood glucose levels to maintain glucose homeostasis.^[40]^ Besides, the PI3K/Akt signal pathway and its downstream molecules play a key role in the treatment of obesity and type 2 diabetes.^[41]^ Moreover, studies have shown that reducing inflammation in mice can improve outcomes in type 2 diabetes by activating insulin signaling in the liver and overcoming damage to the PI3K/Akt insulin signal pathway.^[42]^ Activation of the PI3K/Akt signal pathway could coordinate the response of different metabolic and inflammatory signals in macrophages, and it played an essential role in the physiology of adipocytes including glucose homeostasis and cell differentiation.^[43]^ Therefore, the PI3K/Akt signal pathway played an important role in regulating insulin resistance and hyperglycemia in type 2 diabetes. A previous study had claimed that the AGE-RAGE signal pathway could affect the severity of diabetic complications by mediating vascular calcification.^[44]^ In addition, the AGE-RAGE signal pathway was also closely related to the development of fatty liver disease induced by type 2 diabetes and early diabetic retinopathy.^[45]^ It has been reported that astragalus membranaceus could treat oxidative stress, angiogenesis, and inflammation in diabetic complications through the AGE-RAGE signal pathway.^[46]^ Therefore, we infer that the PI3K-Akt signaling pathway and AGE-RAGE signaling pathway in diabetic complications could serve as key signal pathways for SMYAD to treat DFU.

There may be some limitations to this study. Firstly, the network pharmacology-related data and gene expression data used in the study were mainly obtained from public databases. Secondly, the conclusions drawn in the study were inferred and simulated through software, lacking further validation through experiments in vivo or in vitro. However, this study not only supplemented the shortcomings of SMYAD in the treatment of diabetic foot but also explored its mechanism of action.

In summary, we utilized network pharmacology methods and molecular docking to explore the mechanism of action of the key active ingredients of SMYAD on the key targets of DFU. SMYAD may primarily treat DFU through the PI3K-Akt signaling pathway and AGE-RAGE signaling pathway in diabetic complications.

## Author contributions

**Conceptualization:** Qiang Zhang.

**Data curation:** Qiang Zhang, Jing Li.

**Formal analysis:** Qiu-ying Zhang.

**Investigation:** Ji Guan.

**Methodology:** Lin Wang.

**Supervision:** Qiang Zhang.

**Writing – original draft:** Qiang Zhang, Jing Li, Lin Wang, Qiu-ying Zhang, Ji Guan.

**Writing – review & editing:** Qiang Zhang.
